# Socioeconomic inequalities in adverse pregnancy outcomes in India: 2004–2019

**DOI:** 10.1371/journal.pgph.0003701

**Published:** 2024-09-18

**Authors:** Caroline M. Joyce, Deepti Sharma, Arnab Mukherji, Arijit Nandi

**Affiliations:** 1 Department of Epidemiology, Biostatistics, and Occupational Health, School of Population and Global Health, McGill University, Montreal, Quebec, Canada; 2 Center for Public Policy, Indian Institute of Management Bangalore, Bengaluru, Karnataka, India; 3 Institute for Health and Social Policy, McGill University, Montreal, Quebec, Canada; PLOS: Public Library of Science, UNITED STATES OF AMERICA

## Abstract

Although India has made substantial improvements in public health, it accounted for one-fifth of global maternal and neonatal deaths in 2015. Stillbirth, abortion, and miscarriage contribute to maternal and infant morbidity and mortality. There are known socioeconomic inequalities in adverse pregnancy outcomes. This study estimated changes in socioeconomic inequalities in rates of stillbirth, abortion, and miscarriage in India across 15 years. We combined data from three nationally representative health surveys. Absolute inequalities were estimated using the slope index of inequality and risk differences, and relative inequalities were estimated using the relative index of inequalities and risk ratios. We used household wealth, maternal education, and Scheduled Caste and Scheduled Tribe membership as socioeconomic indicators. We observed persistent socioeconomic inequalities in abortion and stillbirth from rates of 2004–2019. Women at the top of the wealth distribution reported between 2 and 5 fewer stillbirths per 1,000 pregnancies over the study time period compared to women at the bottom of the wealth distribution. Women who completed primary school, and those at the top of the household wealth distribution, had, over the study period, 5 and 20 additional abortions per 1,000 pregnancies respectively compared to women who did not complete primary school and those at the bottom of the wealth distribution. Women belonging to a Scheduled Caste or Scheduled Tribe had 5 fewer abortions per 1,000 pregnancies compared to other women, although these inequalities diminished by the end of the study period. There was less consistent evidence for socioeconomic inequalities in miscarriage, which increased for all groups over the study period. Despite targeted investments by the Government of India to improve access to health services for socioeconomically disadvantaged groups, disparities in pregnancy outcomes persist.

## Introduction

India has made substantial improvements in public health over the last few decades, including significant reductions in neonatal and maternal mortality [1]. Despite these improvements, India accounted for an estimated one-fifth of all global maternal and early neonatal deaths in 2015 [2, 3]. India is not on track to meet the United Nations (UN) Sustainable Development Goal (SDGs) 3 by 2030 [3, 4], including the subgoals to lower rates of maternal and infant mortality [4]. Stillbirth (non-live births ≥ 28 weeks of pregnancy) and miscarriage (pregnancy loss < 28 weeks) have been shown to increase the likelihood of maternal morbidity and mortality [5], and prior stillbirth is a key predictor of fetal and neonatal death in subsequent pregnancies [6]. These adverse outcomes are therefore important indicators of a country’s progress on SDG 3. India accounts for 19% of births worldwide but 22.6% of the global burden of stillbirths, more than any other country [7]. India also has high rates of miscarriage [8], with recent research showing that rates of recurrent miscarriage are higher in India compared to high-income countries [9]. Reducing pregnancy losses is critical to accelerating decreases in rates of maternal and neonatal mortality [10], and achieving India’s SDG targets.

There are multiple factors that can contribute to stillbirth and miscarriage. The World Health Organization (WHO) states that access to quality family planning–including modern contraception and safe induced abortion–is critical to preventing unplanned pregnancies, and lowering rates of miscarriage and stillbirth [11]. The medical procedure used for abortion (both surgery and medication) is also often a necessary treatment for miscarriage [12]. India legalized abortion in 1971 [13] and granted widespread access to medication-induced abortion in 2003 [14], and therefore has comparatively liberal abortion laws. While surgical abortion is technically free at public facilities, women may be subject to other costs [15], including the direct costs of the anesthesia and medication necessary for the procedure [16], along with indirect costs such as missing work or transportation to the facility [17]. The drugs required for medication-induced abortion are not free, and women must pay out-of-pocket to access this care [15, 18]. Lack of access to quality and safe abortion is a primary risk factor for miscarriage and stillbirth.

Preventable risk factors for experiencing a stillbirth or miscarriage include lack of antenatal care (ANC), giving birth without a skilled birth attendant (SBA), and receiving inadequate nutrition during pregnancy [19]. Approximately 40% of stillbirths occur during prolonged labor, which is more common among women who did not receive quality ANC or live far from emergency obstetric care [20]. Poorer women in India, including women with less formal schooling or illiterate women, are at greater risk for stillbirth and miscarriage because they are less likely to receive ANC, deliver with a SBA [21, 22], and a diet that does not met core nutritional needs [23–25]. Additionally, women belonging to designated Scheduled Castes and Scheduled Tribes, defined by the UN as groups of people that, both historically and currently, endure systemic discrimination impacting all levels of life–especially health [26]–have been shown to have higher rates of adverse pregnancy and neonatal outcomes [10]. Socioeconomically disadvantaged women also have less access to modern contraceptives and safe abortion services [27], often due to financial barriers that prevent women from accessing this type of care. Without access to quality family planning, including abortion, women of lower socioeconomic status are more likely to experience unplanned pregnancies and receive inadequate ANC.

There are limitations to existing research for measuring socioeconomic disparities in rates of stillbirth, abortion, and miscarriage in India. Most research has been cross-sectional and collapses across years[8, 10, 25, 28, 29], including research using Demographic Health Survey (DHS) data [30–32], with few studies examining year-over-year trends or multiple pregnancy outcomes nationally [33]. Similarly, few studies have examined socioeconomic disparities from a pan-Indian perspective [10, 25]. A comprehensive national analysis of trends in levels and disparities in pregnancy outcomes is needed to measure progress towards the SDGs.

This study aims to provide robust, country-wide estimates of year-over-year trends in socioeconomic inequalities in adverse pregnancy outcomes. In particular, we used three nationally representative surveys of ever married women in India to estimate the association between socioeconomic position, measured by the household wealth index, women’s educational attainment, and membership in a Scheduled Caste or Scheduled Tribe, and rates of stillbirth, abortion, and miscarriage from 2004–2019.

## Methods

### Data sources

Data were derived from three district-representative household surveys with similar target populations, sampling designs, and survey instruments that were conducted by the Ministry of Health and Family Welfare (MoHFW): (1) District Level Household Surveys (DLHS) rounds 3 and 4 [34, 35]; (2) National Family Health Survey (NFHS) rounds 4 and 5 [31, 36]; and (3) the Annual Health Survey (AHS) of India [37]. These data have been pooled previously to facilitate cross-sectional and longitudinal analyses [38–43]. The DLHS, AHS, and NFHS provide district-representative snapshots of the target population of ever-married women between 15 and 49 years of age in nearly all states and territories during the study period from 2004–2019.

We pooled the information provided by women in each survey on their reproductive histories to construct a panel of pregnancies and corresponding pregnancy outcomes (i.e., live birth, miscarriage, abortion, or stillbirth) over the study period. For comparability across surveys, we included only the most recent reported pregnancy. In total, we recorded 2,005,290 pregnancies that resulted in live births, still births, miscarriages, or abortions between 2004 and 2019 (**S1 Fig**). Technical details for each survey are available elsewhere [30, 31, 34, 44].

### Measures

Our primary outcomes of interest were self-reported stillbirth, abortion, and miscarriage. In the DLHS and AHS surveys women were asked to report all pregnancies and their outcomes in the last three years. In the NFHS, the contraceptive calendar was used to self-report reproductive health outcomes (contraception, pregnancies, births) for each month of the past five years. Following DHS guidelines, spontaneous abortions before the seventh month (28 weeks) of pregnancy were defined as miscarriages, and those that occurred during or after the seventh month (28 weeks) of pregnancy were defined as stillbirths [45, 46]. We used this information to define three binary outcome variables for each participant’s most recent pregnancy: 1) stillbirth; 2) induced abortion; or 3) miscarriage (also referred to as spontaneous abortion).

Household and respondent-level socioeconomic characteristics that matched across surveys were used to measure social inequalities. We used information on reported household assets [47] to create a continuous wealth index. This index was used to create a continuous rank measure of the woman’s socioeconomic position relative to others in the same survey year. Further details on the creation of the wealth index are available in **S1 Text**, **and Table A and Fig A in S1 Text**. Education was dichotomized based on whether the respondent completed a primary education (≥ 8 years of schooling) or not [25, 48, 49]. Two dichotomous variables were created for whether the respondent reported belonged to a Scheduled Caste or a Scheduled Tribe. The comparison group for these analyses were women who did not belong to a Scheduled Caste or Scheduled Tribe, which is labeled as “None”. Age was included as a covariate, and state of residence and year of outcome assessment were included as fixed effects. To examine changes over time, we estimated rates by two-year intervals.

### Statistical analyses

We estimated social inequalities in rates of stillbirth, abortion, and miscarriage. For binary socioeconomic factors (i.e., education and belonging to a Scheduled Caste or Scheduled Tribe) we used generalized linear models (GLMs) with a Gaussian distribution and an identity link to estimate associations on the risk difference (RD) scale and with a Poisson distribution and a log link to estimate associations on the risk ratio (RR) scale [50–53].

For the continuous household wealth index, we measured social inequalities on the relative scale using the relative index of inequality (RII) and on the absolute scale using the slope index of inequality (SII). The RII and SII are regression based measures that account for the relation between a socioeconomic indicator and outcome across the entire distribution of the socioeconomic gradient, and are recommended for examining inequalities across groups or time [54, 55]. The RII and SII are defined as the ratio and difference of the risk of the health outcome, respectively, comparing those in the highest vs. the lowest socioeconomic position.

Pregnancies were assigned the respondent’s wealth score and ranked from lowest to highest position in the socioeconomic gradient. For descriptive statistics, these rankings were then divided into quintiles. For regression estimates, each observation was assigned a ranking, r_it_, based on its position in the cumulative distribution of the socioeconomic indicator.

The RII and SII were estimated using the following GLM:

$$f(\pi_{it})=\beta_{0}+\beta_{1}r_{it}+\Sigma_{k}\delta_{k}X_{it}+\varepsilon ,$$ (1)

where π = E(Y_it_) = P(Y_it_ = 1) for the binary outcome of interest (i.e., stillbirth, abortion, miscarriage), r_it_ is the fractional rank for observation i in year t, and X_it_ is a vector of k covariates. A linear probability model with an identity link function [i.e., f(π) = π)] was used to calculate the SII, presented as the difference in the risk of the outcome per 1,000 pregnancies. To calculate the RII, we fitted a Poisson regression model with a log link function [i.e., f(π) = log(π)] and robust variance estimators [56]. The RII and SII are defined as the ratio and difference of the risk of the health outcome, respectively, comparing those at the top (r_i_ = 1) and bottom (r_i_ = 0) of the socioeconomic gradient [57].

To enhance the comparability of our estimates, we adjusted for a vector of k covariates, X_k_, that may vary across groups and time, including age of the mother, state of residence, and urban vs. rural residence. Results were stratified by year. As survey-specific sampling weights are relative weights and do not provide valid estimates when surveys are pooled, the weights from each survey were de-normalized [58]. We report inequality measures with corresponding robust 95% confidence intervals. All analyses were run using the survey package [59] in R Version 4.1.3 to account for the complex survey design [60].

### Ethics statement

This research study was approved by the McGill University Faculty of Medicine and Health Sciences Institutional Review Board approval number A05-E24-21B (21-05-038). This research uses anonymized survey data collected by outside organizations. The researchers in this study did not have any role in collecting data. All surveys obtained informed consent for each participant [35, 36, 61–63]. Ever-married women aged 15 and older gave consent for themselves, with 15 considered the minimum age to give informed consent [64].

## Results

Respondent characteristics are shown in **Table 1**. The sample included the 2,005,290 most recent pregnancies from surveyed women. The median age of women at time of survey was 24 (IQR = 7) years, with 50% having completed primary school (≥ 8 years of school). The majority of respondents (81%) lived in a rural location and, and a minority (34%) reported belonging to either a Scheduled Caste or Scheduled Tribe.

**Table 1:** Socio-demographic information on respondents.

Variable	N = 2,005,290^1^
Age	24 (7)
Missing	6,490
Rural / Urban	
Urban	375,453 (19%)
Rural	1,629,837 (81%)
Member of Scheduled Caste or Scheduled Tribe	
None	1,305,093 (66%)
Scheduled Caste	382,551 (19%)
Scheduled Tribe	292,667 (15%)
Missing	24,979
Completed Primary School	954,921 (50%)
Missing	108,309

^1^Median (IQR); n (%)

### Descriptive trends

**Fig 1:**
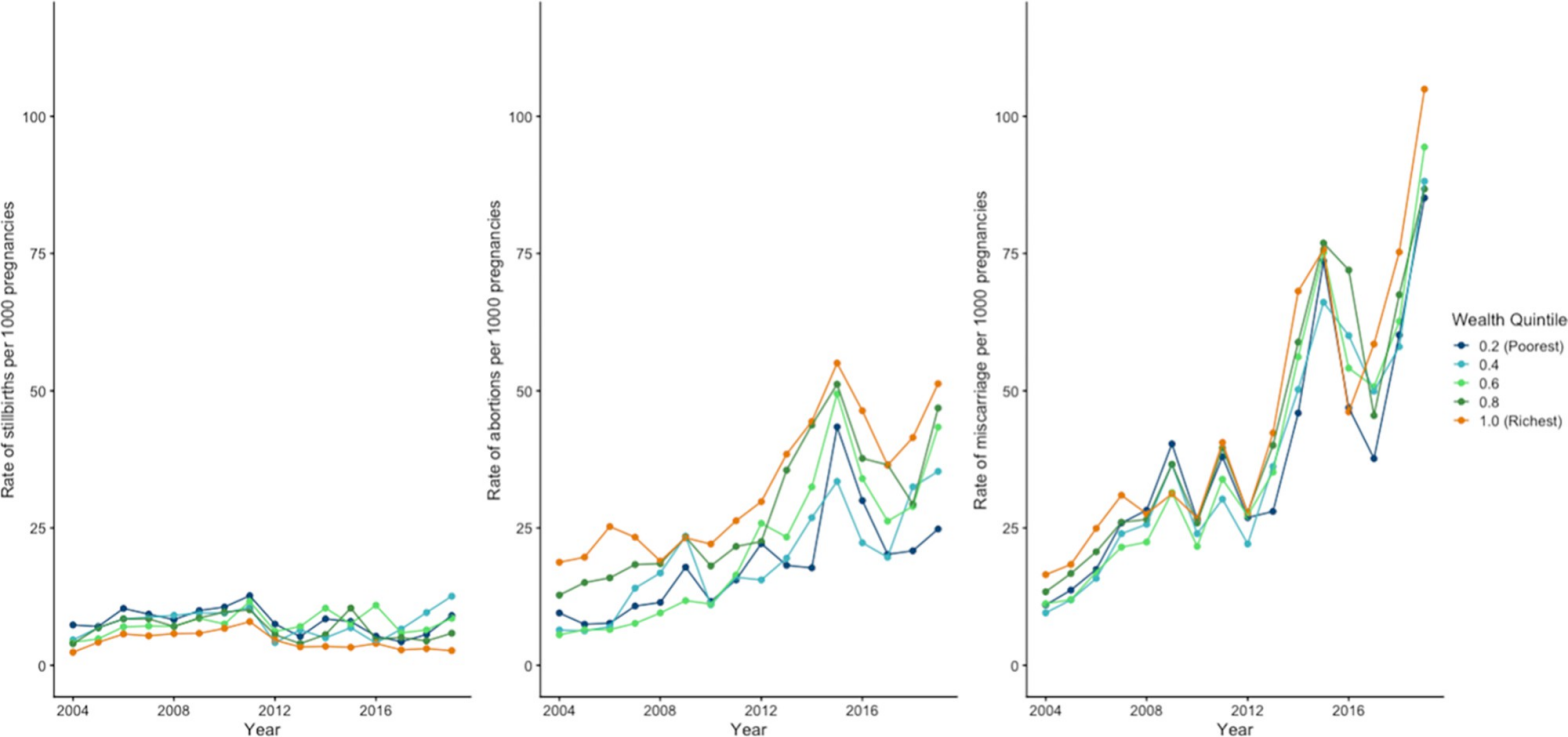
Rates of stillbirths, abortions, and miscarriages per 1,000 pregnancies by wealth quintile, 2004–2019.

Across all socioeconomic characteristics, there were increases in rates of abortion and miscarriage after 2008. **Fig 1** shows annual rates of stillbirth, abortion, and miscarriage per 1,000 pregnancies for each wealth quintile. There was a consistent social gradient in rates of stillbirth, with the lowest rates among those in the highest wealth quintiles across almost all study years. Conversely, rates of abortion were consistently higher in the highest wealth quintile, although in later years we observed increases in the lower wealth quintiles. There was no discernible wealth-based gradient for miscarriage, although we observed a larger increase in rates of miscarriage among women in the lowest wealth quintile. This trend was seen across states in the study sample (**S2 Fig**).

**Fig 2:**
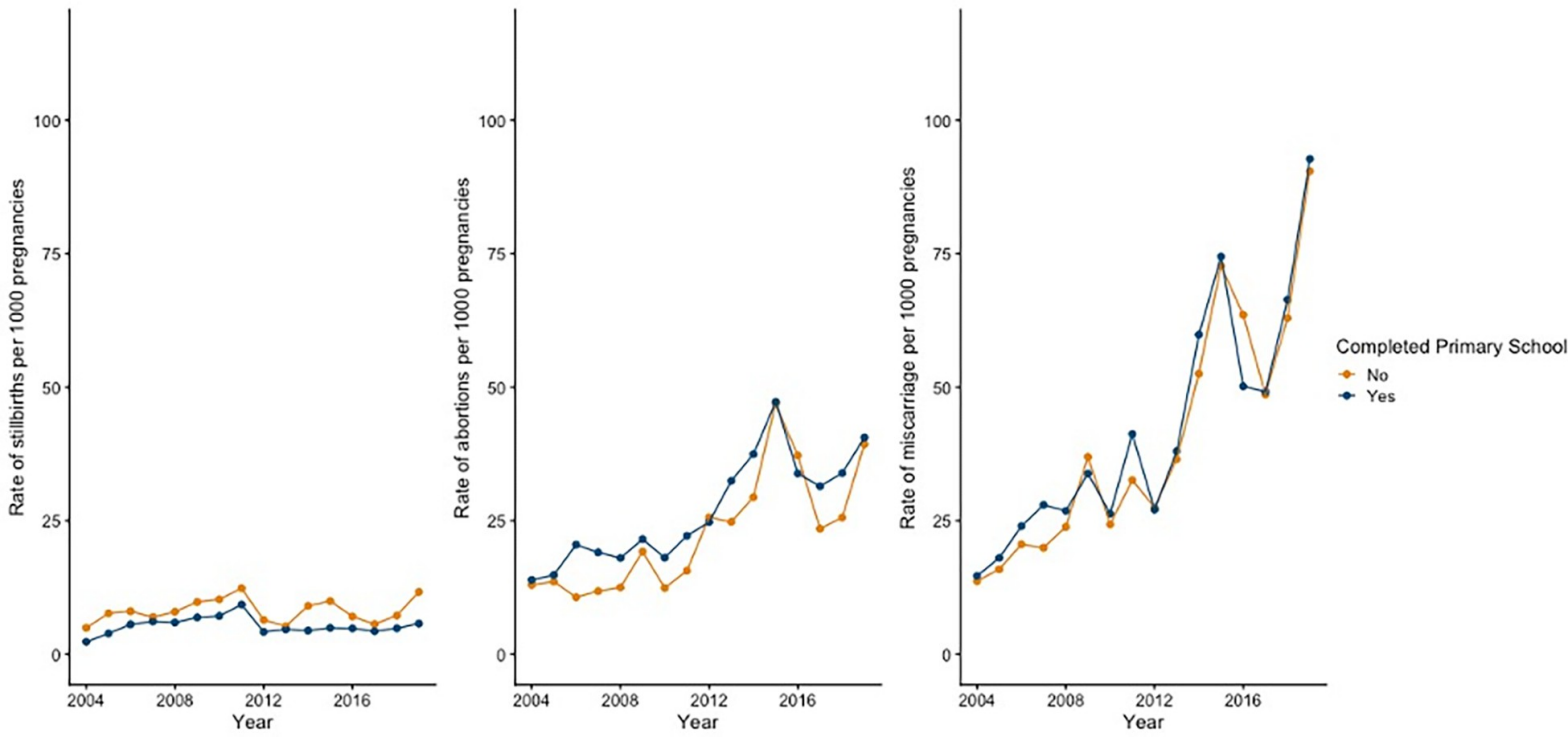
Rates of stillbirths, abortions, and miscarriages per 1,000 pregnancies by educational level, 2004–2019.

**Fig 3:**
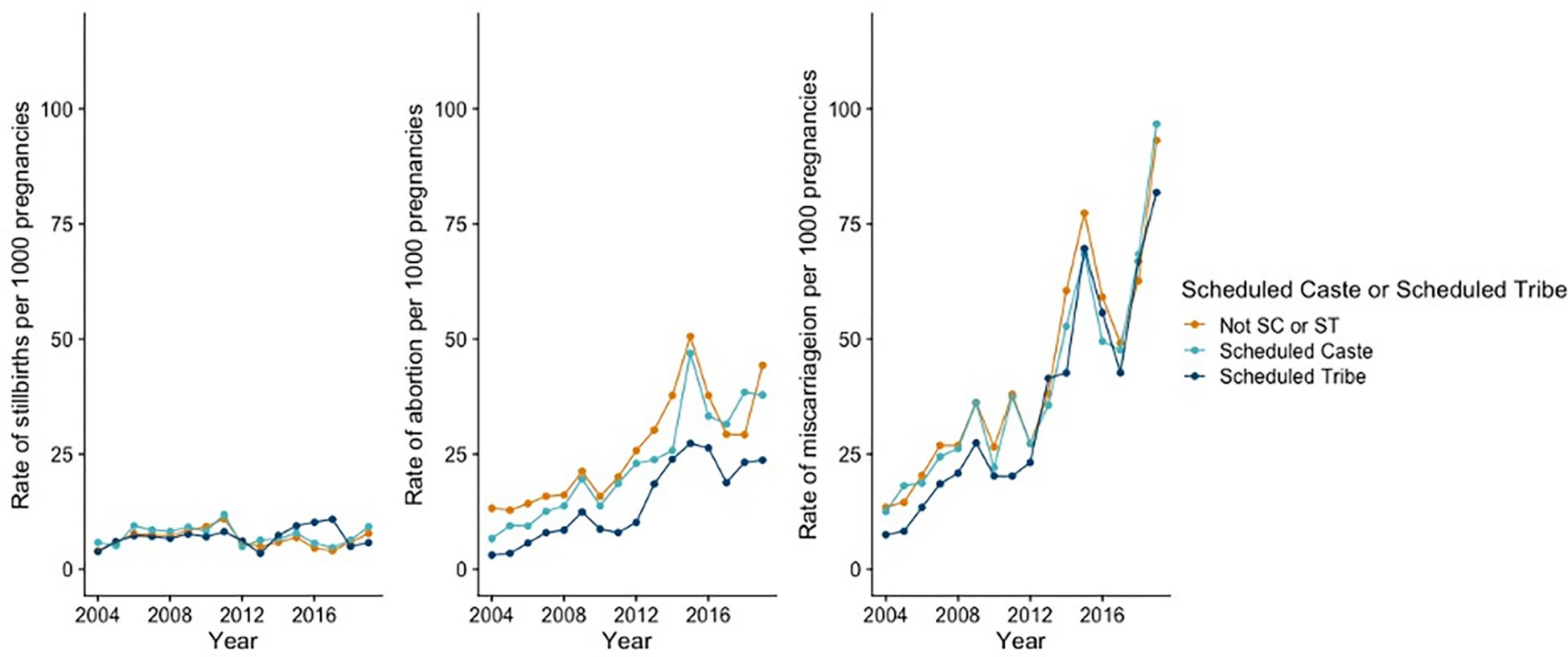
Rates of stillbirth, abortion, and miscarriage per 1,000 pregnancies by Schedule Caste/Scheduled Tribe group, 2004–2019.

**Fig 2** shows trends by whether the respondent completed primary school education. Rates of stillbirth were higher among women without a primary school education for each year surveyed. For abortion, rates were higher among women with a primary education between 2004–2011, however, the gap narrowed after 2012. Rates of miscarriage were similar across the two groups. Finally, **Fig 3** shows outcomes by whether the respondent belonged to a Scheduled Caste, a Scheduled Tribe, or neither. Rates of stillbirths and miscarriages were similar among all three groups, although rates of stillbirth increased among women belonging to a Scheduled Tribe relative to the other groups. Additionally, rates of abortion were lowest for women who belonged to a Scheduled Tribe.

### Social inequalities measured by the wealth index

**Fig 4:**
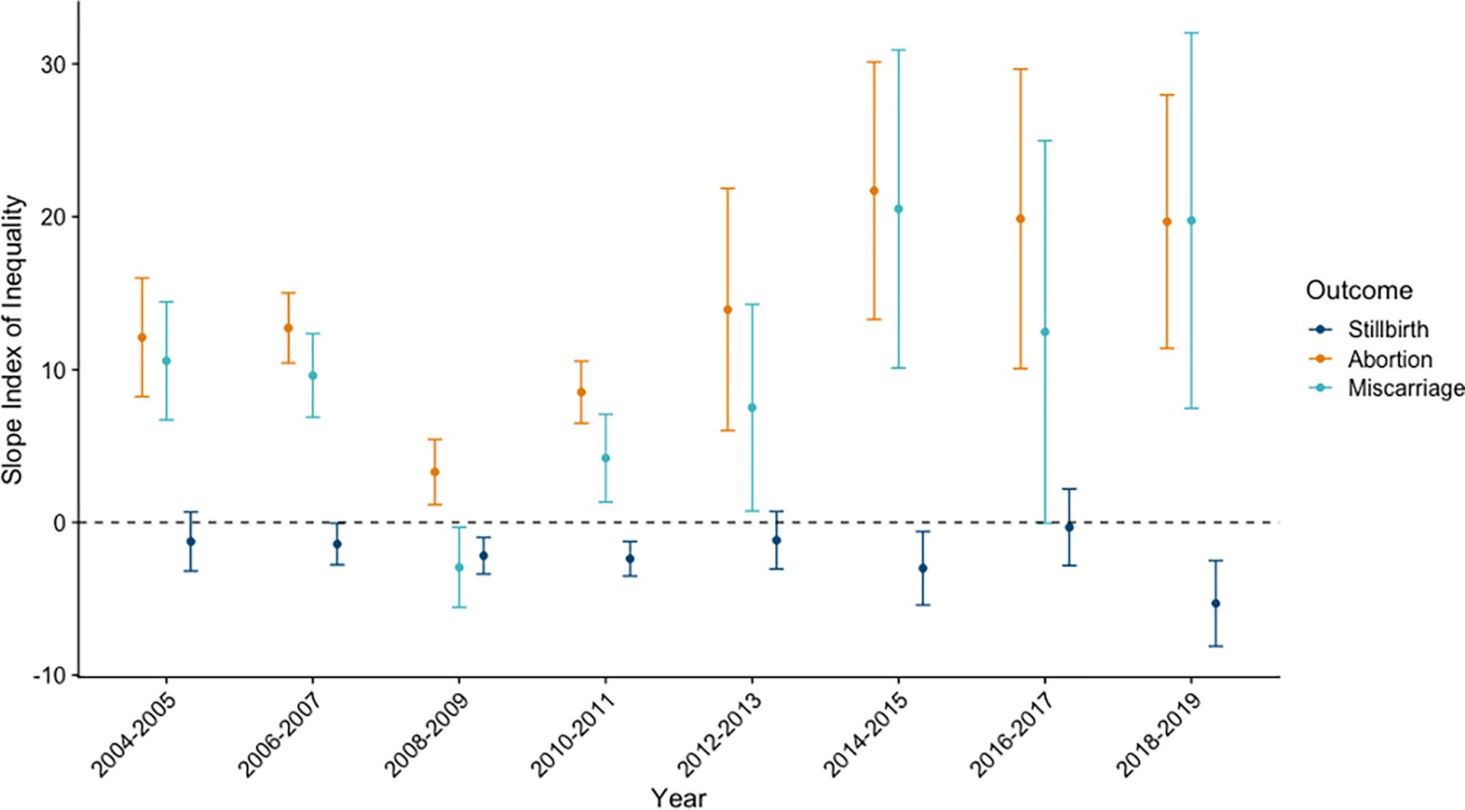
Slope index of inequality for measuring inequalities by household wealth in stillbirth, abortion, and miscarriage; India; 2004–2019.

We observed wealth-based inequalities for all three outcomes. Estimates of the SII stratified by years are shown in **Fig 4**. Differences in rates of stillbirth between the top and bottom of the wealth distribution were observed in all years except 2004–2005, 2012–2013, and 2016–2017. Rates were consistent across the study period, with SII estimates of -1.4 (95% CI: -2.8, -0.6) in 2006–2007 and -5.3 (95% CI: -8.1, -2.5) in 2018–2019 (**Fig 4**). This indicates that over the study period women at the top of the wealth distribution experienced between 2 and 5 fewer stillbirths per 1,000 pregnancies compared to women at the bottom of the wealth distribution. A similar pattern was observed on the relative scale, with an RII of 0.8 (95% CI: 0.7, 0.9) in 2006–2007 and 0.4 (95% CI: 0.3, 0.6) in 2018–2019 (**S3 Fig**).

Absolute rates of abortion for women at the top of the wealth distribution were higher for all study years. The SII was 12.1 (95% CI: 8.2, 15.9) in 2004–2005 and 19.7 (95% CI: 11.4, 27.9) in 2018–2019, indicating that women at the top of the wealth distribution reported 12.1 and 19.7 more abortions per 1,000 pregnancies, respectively, compared to women at the bottom of the wealth distribution (**Fig 4**). On the relative scale, the RII was higher in 2004–2005 [RII = 3.5 (95% CI: 2.5, 4.9)] compared to 2018–2019 [RII = 1.6 (95% CI: 1.2, 1.9)], since abortion rates increased over the study period (**S3 Fig**). For miscarriage, women at the top of the wealth distribution reported 10.6 (95% CI: 6.7, 14.4) more miscarriages per 1,000 pregnancies in 2004–2005 and 19.8 (95% CI: 7.5, 32.0) more miscarriages per 1,000 pregnancies in 2018–2019, compared to those at the bottom of the wealth distribution (**Fig 4**). On the relative scale, the RII was 1.8 (95% CI: 1.4, 2.3) in 2004–2005 and 1.3 (95% CI: 1.1, 1.5) in 2018–2019 (**S3 Fig**). For all outcomes there was a significant interaction on the absolute scale between individual rank and year (p < 0.05), indicating heterogeneity in the magnitude of the inequality over the study period.

### Education

**Fig 5:**
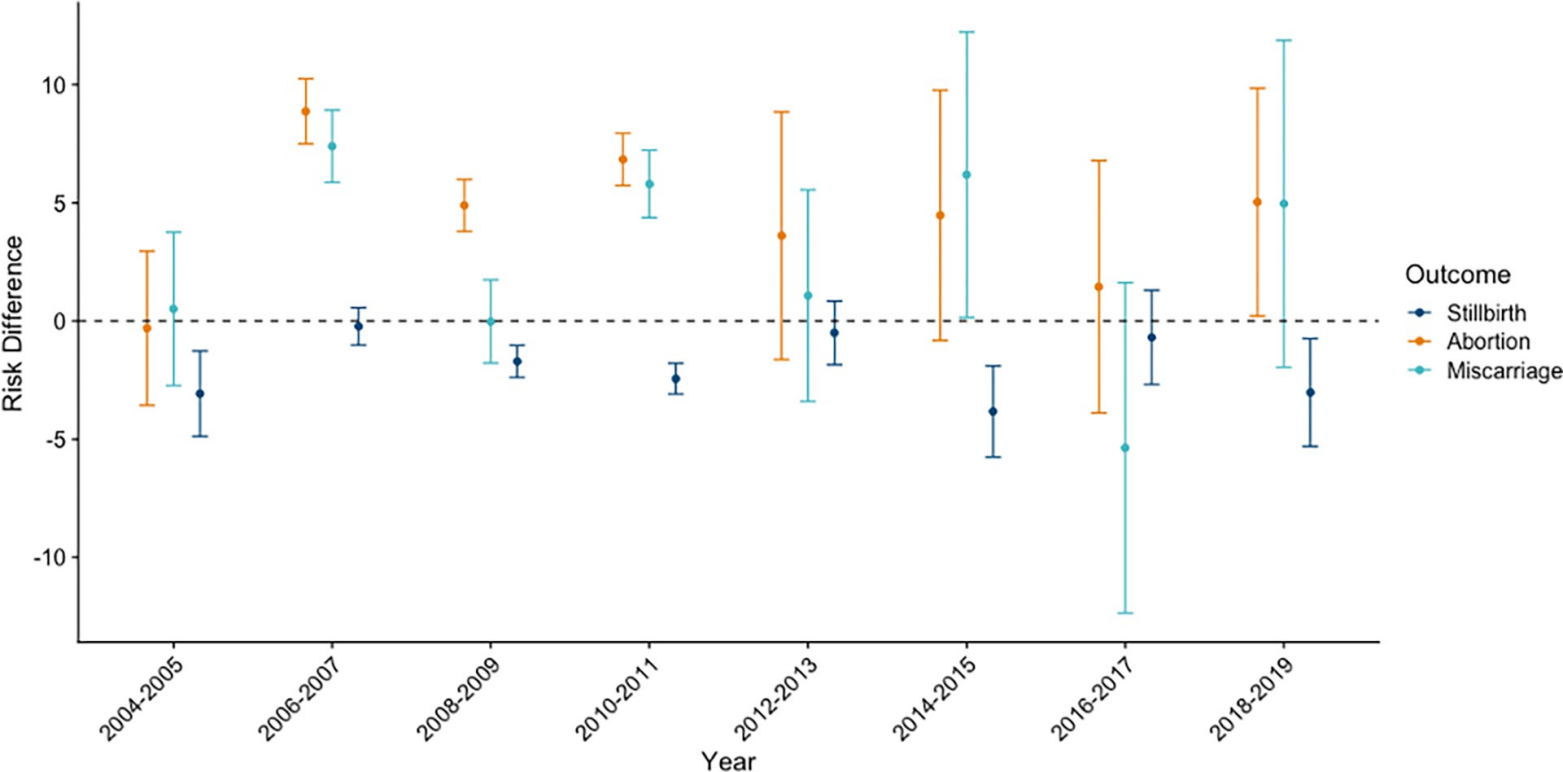
Risk differences in stillbirth, abortion, and miscarriage by primary education attainment; India; 2004–2019.

Similar social inequalities in pregnancy outcomes were observed by educational attainment (**Fig 5**). Across most study years, rates of stillbirth were lower among women who completed primary school compared to those who did not. In 2004–2005, women who completed primary school had an absolute risk of -3.1 (95% CI: -4.9, -1.3) stillbirths per 1,000 pregnancies, compared to women who did not, which remained fairly consistent across study years. However, we did observe a significant interaction between education and year (p < 0.05) on the absolute scale, indicating heterogeneity in the magnitude of the inequality over the study period. On the relative scale, women who completed primary school had a relative risk for stillbirth of 0.5 (95% CI: 0.4, 0.7) in 2004–2005 and 0.7 in 2018–2019 (95% CI: 0.5, 0.9) (**S4 Fig**). Rates of abortion were higher among women who completed primary school compared to those who did not for many, but not all, years. In 2004–2005 there was no observed difference in the risk of abortion for women by educational attainment (RD = -0.3, 95% CI: -3.6, 2.9); however, the RD was positive in later years, with an estimate of 5.0 (95% CI: 0.2, 9.9) additional abortions per 1,000 pregnancies at the end of the study period in 2018–2019 (**Fig 5**). The corresponding estimates on the relative scale were RR = 1.0 (95% CI: 0.8, 1.3) in 2004–2005 and RR = 1.2 (95% CI: 1.0, 1.4) in 2018–2019 (**S4 Fig**). Education-based inequalities in miscarriage were less consistent over the study period (**Fig 5** and **S4 Fig**).

### Scheduled Caste or Scheduled Tribe

**Fig 6:**
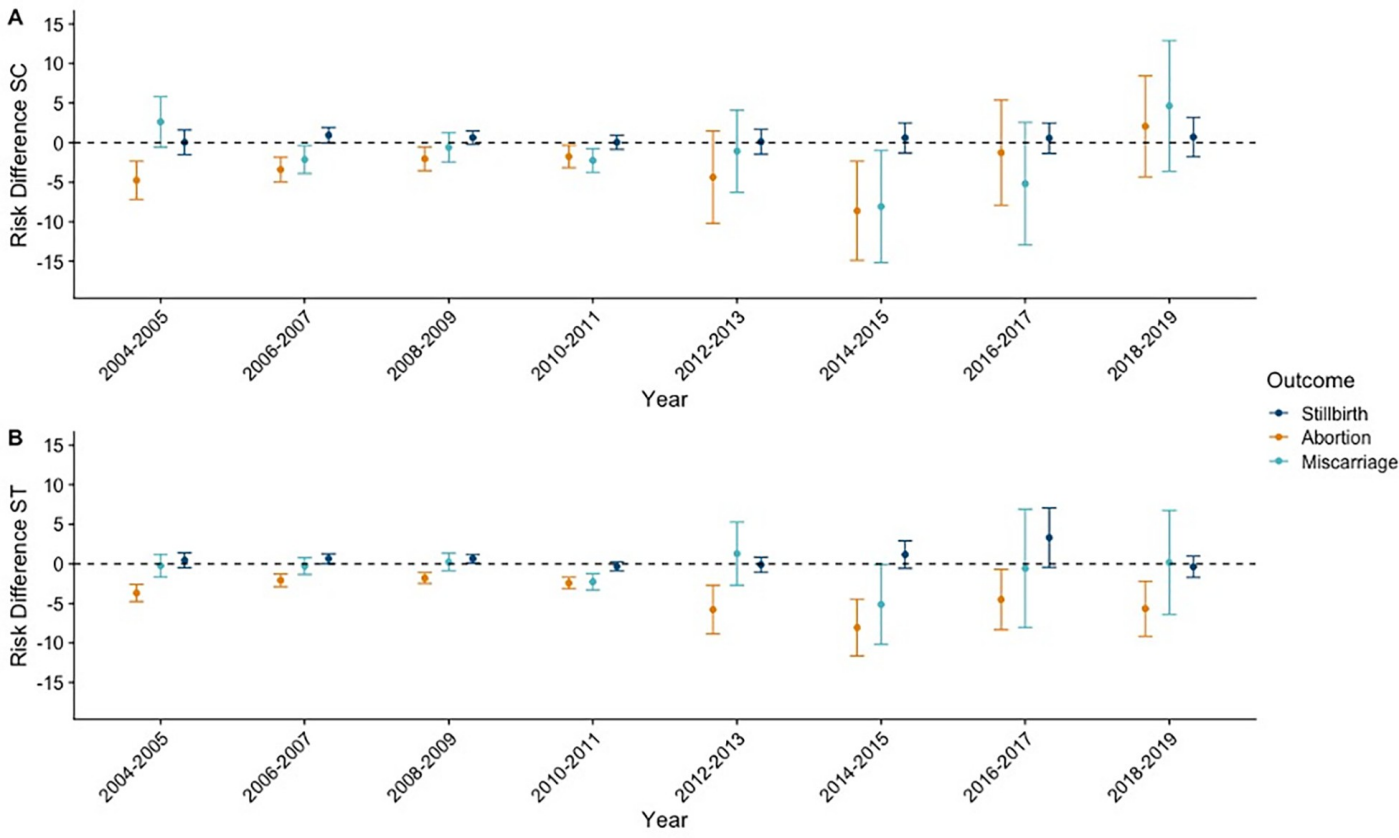
Risk differences in stillbirth, abortion, and miscarriage by Scheduled Caste or Scheduled Tribe status; India; 2004–2019.

Differences in pregnancy outcomes by membership in a Scheduled Caste or Scheduled Tribe were less apparent. For most years, there were little evidence of differences in the risk of stillbirth and miscarriage for women who belonged to Scheduled Caste on the absolute or relative scales (**Fig 6** and **S5 Fig**). In 2004–2005, women belonging to a Scheduled Caste reported 4.8 fewer abortions per 1,000 pregnancies (95% CI -7.2, -2.4) compared to women who did not belong to a Scheduled Caste or Scheduled Tribe. However, by 2018–2019 the RD was 2.1 (95% CI: -4.3, 8.4) (**Fig 6**), with similar patterns on the RR scale (**S5 Fig**). We did not observe consistent inequalities in risks of stillbirth or miscarriage for women who belonged to a Scheduled Tribe across study years (**Fig 6** and **S5 Fig**). However, across all study years, the risk of abortion was lower for women belonging to Scheduled Tribe compared to women who were not in a Scheduled Caste or Scheduled Tribe, with an RD of 3.7 fewer abortions per 1,000 pregnancies (95% CI: -4.8, -2.6) in 2004–2005, and an RD of 5.7 fewer abortions per 1,000 pregnancies (95% CI: -9.2, 2.2) in 2018–2019, with relative estimates shown in **S5 Fig**.

## Discussion

This study provides national estimates of socioeconomic inequalities in adverse pregnancy outcomes across a 15-year time span. We observed persistent socioeconomic inequalities in rates of abortion and stillbirth in India during the study period from 2004–2019. The magnitude of the inequalities varied according to the socioeconomic indicator and outcome. Across most study years, women at the top of the household wealth distribution and women who completed primary school were more likely to report an abortion and less likely to experience a stillbirth compared to women at the bottom of the wealth distribution and women who had not completed primary school. Women belonging to Scheduled Tribes were less likely to have an abortion compared to women who did not belong to either Scheduled Caste or Scheduled Tribe. There was less consistent evidence for socioeconomic inequalities in miscarriage, for which there were stronger positive trends for all groups over the study period and greater between year variability in rates. There were substantial increases in rates of abortion and miscarriage, with an acceleration in rates across all socioeconomic groups after 2008.

Our research follows prior work showing socioeconomic disparities in adverse pregnancy outcomes in India. This is in spite of targeted investment, such as conditional cash transfer programs like Janani Suraksha Yojana (JSY), by the GoI specifically aimed to improve pregnancy outcomes [65]. Higher abortion rates among women of higher socioeconomic status have been documented by previous studies [15, 66]. Our results are also in line with the national estimates obtained from National Family Health surveys, India’s version of the Demographic Health Survey [30–32]. There were also results that differed from previous literature. Previous research has showed that higher education was associated with higher risk of miscarriage [8], and that maternal education is the strongest predictor of induced abortion likely due to both access and resources for payment [67]. Prior work also shows disparities in access to, and utilization of, maternal and reproductive health services by women belonging to a Scheduled Caste or Scheduled Tribe [68]. We observed disparities in miscarriage and abortion, though not in the later study years. We did not observe disparities in rates of stillbirth. We observed that belonging to the highest quintile of the household wealth distribution was a stronger and more consistent predictor of lower stillbirth and higher abortion rates than completing primary education. This may be because greater household wealth and attendant material resources facilitate access to essential reproductive services such as abortion or antenatal care, which are important mechanisms for preventing stillbirth [15, 69]. Recent research has shown that rates of stillbirth and miscarriage in India are increasing, with a corresponding decrease in rates of livebirths [70]. Our analyses indicate that these observed increases are not equal across socioeconomic groups.

There are several limitations to the reported research. First, all outcomes were self-reported and subject to measurement error, including recall bias. However, these surveys use well-validated instruments [71, 72] and we restricted our sample to the most recent pregnancy, which should reduce recall bias [44]. Secondly, the AHS focused on nine states with disproportionately worse maternal and infant health outcomes, resulting in a larger number of observations from these states between 2007–2011. Additionally, pregnancy information from some years (e.g., 2007) was available from multiple surveys (e.g., AHS and DLHS-3), resulting in uneven sample sizes per year and higher levels of precision in the earlier part of the study period. However, before pooling data from multiple surveys, we de-normalized each survey’s sampling weights based on the proportion of women aged 15–49 in each state, available from the Census [73, 74], who were sampled by the survey, using the de-normalization of standard weights approach described in the DHS Sampling and Household Listing Manual [75].Thirdly, abortion may have been underreported or misclassified as miscarriage, and this error may have been differential by socioeconomic status. Due to social stigma and bias, it is likely that some abortions were self-reported as miscarriages, as abortion has been found to be underreported among Indian women of lower socioeconomic status [15]. This misclassification is common when measuring abortion–particularly for self-managed medication abortion [76], and is a salient issue in India, where combating gender-biased sex selection by abortion is a top priority for the national government [77, 78]. All else equal, differential underreporting among socially disadvantaged groups may have resulted in an overestimate of the disparities in reported abortion by household wealth and education reported above.

## Conclusion

Our study provides evidence of persistent socioeconomic inequalities in rates of stillbirth and abortion, despite targeted investments by the GoI to improve access to healthcare among socioeconomically disadvantaged groups. Additionally, we observed pronounced increases in rates of miscarriage across all socioeconomic groups. Further research is needed to investigate these alarming trends, notably the increase in miscarriage rates, and understand the mechanisms underlying social inequalities in pregnancy outcomes, including higher rates of stillbirth and lower rates of abortion among socioeconomically disadvantaged compared to advantaged groups. Further research is also needed to examine state-specific trends in these outcomes.

## Supporting information

S1 Checklist STROBE statement—checklist of items that should be included in reports of observational studies. (PDF)

S1 Fig Flowchart describing creation of analytic samples. (TIFF)

S2 Fig Rates of miscarriage per 1,000 pregnancies by year and state. (TIFF)

S3 Fig Relative index of inequality for relationship between wealth index and stillbirth, abortion, and miscarriage; India; 2004–2019. (TIFF)

S4 Fig Risk ratio measuring the relationship between primary education attainment and stillbirth, abortion, and miscarriage; India; 2004–2019. (TIFF)

S5 Fig Risk ratio measuring relationship between Scheduled Caste or Scheduled Tribe status and stillbirth, abortion, and miscarriage; India; 2004–2019. (TIFF)

S1 Text (DOCX)
