# Supplementary material for: Socioeconomic inequalities in adverse pregnancy outcomes in India: 2004–2019
Source: PLOS Glob Public Health. 2024 Sep 18;4(9):e0003701. doi: 10.1371/journal.pgph.0003701 (PMC11410185; doi:10.1371/journal.pgph.0003701)
Supplement: S1 Checklist — (PDF) [file pgph.0003701.s001.pdf]

STROBE Statement—checklist of items that should be included in reports of observational studies

	Item No	Recommendation
**Title and abstract**	1	(*a*) Indicate the study's design with a commonly used term in the title or the abstract Line 31
		(*b*) Provide in the abstract an informative and balanced summary of what was done and what was found Page 2
**Introduction**		
Background/rationale	2	Explain the scientific background and rationale for the investigation being reported Pages 4 – 6, specifically lines 70 - 115
Objectives	3	State specific objectives, including any prespecified hypotheses Lines 116 - 128
**Methods**		
Study design	4	Present key elements of study design early in the paper Lines 123 - 128
Setting	5	Describe the setting, locations, and relevant dates, including periods of recruitment, exposure, follow-up, and data collection Lines 131 - 145
Participants	6	(*a*) *Cohort study*—Give the eligibility criteria, and the sources and methods of selection of participants. Describe methods of follow-up<br>*Case-control study*—Give the eligibility criteria, and the sources and methods of case ascertainment and control selection. Give the rationale for the choice of cases and controls<br>*Cross-sectional study*—Give the eligibility criteria, and the sources and methods of selection of participants Lines 131 - 145
		(*b*) *Cohort study*—For matched studies, give matching criteria and number of exposed and unexposed<br>*Case-control study*—For matched studies, give matching criteria and the number of controls per case n/a, not matched
Variables	7	Clearly define all outcomes, exposures, predictors, potential confounders, and effect modifiers. Give diagnostic criteria, if applicable Lines 147 - 167
Data sources/ measurement	8*	For each variable of interest, give sources of data and details of methods of assessment (measurement). Describe comparability of assessment methods if there is more than one group Lines 147 - 167
Bias	9	Describe any efforts to address potential sources of bias Lines 140 – 143 and lines 435 - 470
Study size	10	Explain how the study size was arrived at Lines 140 – 145. Supplementary Figure 1.
Quantitative variables	11	Explain how quantitative variables were handled in the analyses. If applicable, describe which groupings were chosen and why Lines 148 - 167
Statistical methods	12	(*a*) Describe all statistical methods, including those used to control for confounding Lines 169 - 202
		(*b*) Describe any methods used to examine subgroups and interactions Lines 169 - 202
		(*c*) Explain how missing data were addressed Supplementary Figure 1
		(*d*) *Cohort study*—If applicable, explain how loss to follow-up was addressed<br>*Case-control study*—If applicable, explain how matching of cases and controls was addressed<br>*Cross-sectional study*—If applicable, describe analytical methods taking account of sampling strategy Lines 196 - 202

(*e*) Describe any sensitivity analyses n/a

Continued on next page

**Results**		
Participants	13*	(a) Report numbers of individuals at each stage of study—eg numbers potentially eligible, examined for eligibility, confirmed eligible, included in the study, completing follow-up, and analysed Supplementary Figure 1
		(b) Give reasons for non-participation at each stage Supplementary Figure 1
		(c) Consider use of a flow diagram Supplementary Figure 1
Descriptive data	14*	(a) Give characteristics of study participants (eg demographic, clinical, social) and information on exposures and potential confounders Table 1
		(b) Indicate number of participants with missing data for each variable of interest Table 1
		(c) *Cohort study*—Summarise follow-up time (eg, average and total amount) n/a
Outcome data	15*	*Cohort study*—Report numbers of outcome events or summary measures over time
		Case-control study—Report numbers in each exposure category, or summary measures of exposure
		Cross-sectional study—Report numbers of outcome events or summary measures Figures 1 – 3 and lines 240 - 258
Main results	16	(*a*) Give unadjusted estimates and, if applicable, confounder-adjusted estimates and their precision (eg, 95% confidence interval). Make clear which confounders were adjusted for and why they were included Figures 4 – 6 and lines 260 - 330
		(*b*) Report category boundaries when continuous variables were categorized Figures 4 - 6
		(*c*) If relevant, consider translating estimates of relative risk into absolute risk for a meaningful time period Absolute estimates given in main text (Figures 4 – 6), relative estimates reported in supplementary figures.
Other analyses	17	Report other analyses done—eg analyses of subgroups and interactions, and sensitivity analyses Lines 260 – 330 give all subgroup analyses
**Discussion**		
Key results	18	Summarise key results with reference to study objectives Lines 3309 - 352
Limitations	19	Discuss limitations of the study, taking into account sources of potential bias or imprecision. Discuss both direction and magnitude of any potential bias Lines 371 - 391
Interpretation	20	Give a cautious overall interpretation of results considering objectives, limitations, multiplicity of analyses, results from similar studies, and other relevant evidence Lines 353 - 370
Generalisability	21	Discuss the generalisability (external validity) of the study results Lines 393 - 401
**Other information**		
Funding	22	Give the source of funding and the role of the funders for the present study and, if applicable, for the original study on which the present article is based Lines 205 – 207 and 405 - 409

*Give information separately for cases and controls in case-control studies and, if applicable, for exposed and unexposed groups in cohort and cross-sectional studies.

Note: An Explanation and Elaboration article discusses each checklist item and gives methodological background and published examples of transparent reporting. The STROBE checklist is best used in conjunction with this article (freely available on the Web sites of PLoS Medicine at http://www.plosmedicine.org/, Annals of Internal Medicine at http://www.annals.org/, and Epidemiology at http://www.epidem.com/). Information on the STROBE Initiative is available at www.strobe-statement.org.
