# Supplementary figures and images for: Socioeconomic inequalities in adverse pregnancy outcomes in India: 2004–2019
Source: PLOS Glob Public Health. 2024 Sep 18;4(9):e0003701. doi: 10.1371/journal.pgph.0003701 (PMC11410185; doi:10.1371/journal.pgph.0003701)

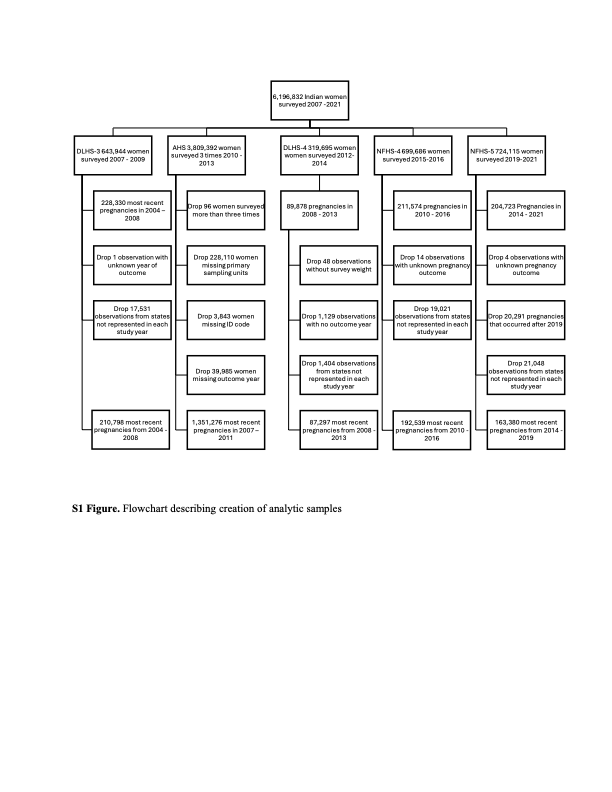

Supplement: S1 Fig — (TIFF) [file pgph.0003701.s002.tiff]

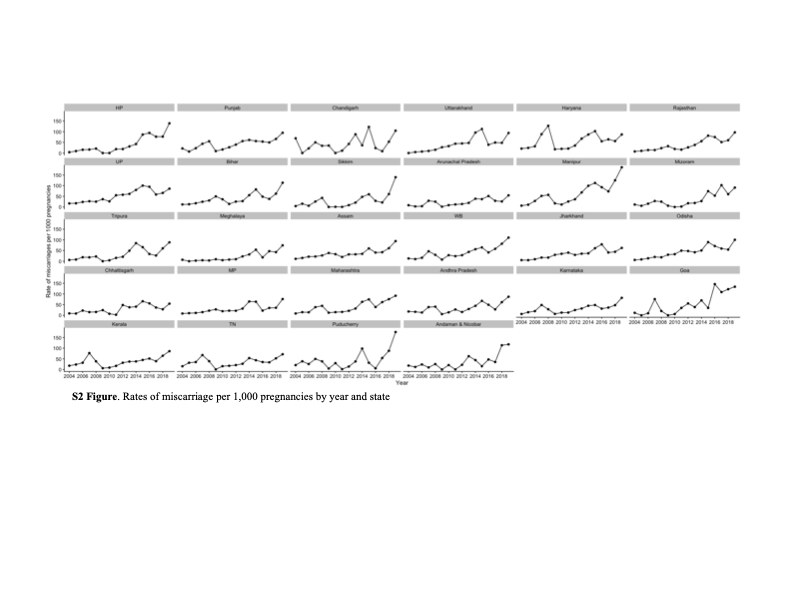

Supplement: S2 Fig — (TIFF) [file pgph.0003701.s003.tiff]

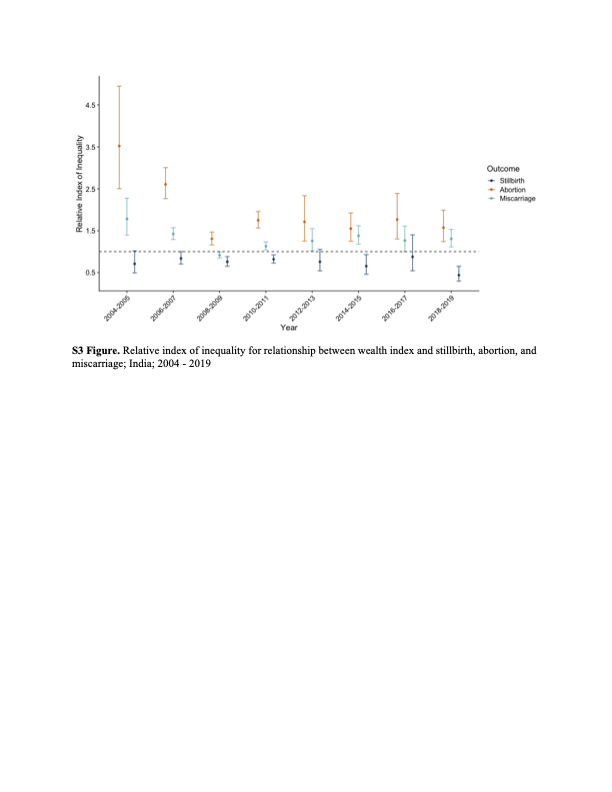

Supplement: S3 Fig — (TIFF) [file pgph.0003701.s004.tiff]

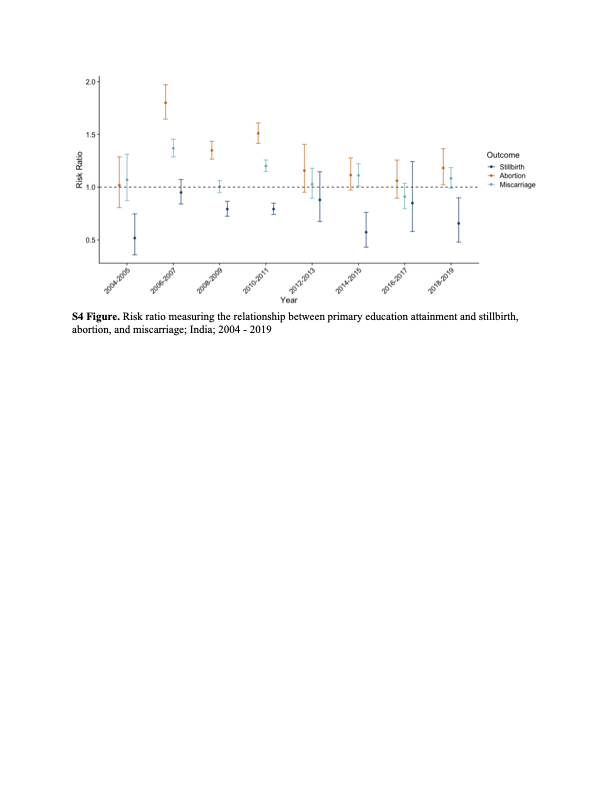

Supplement: S4 Fig — (TIFF) [file pgph.0003701.s005.tiff]

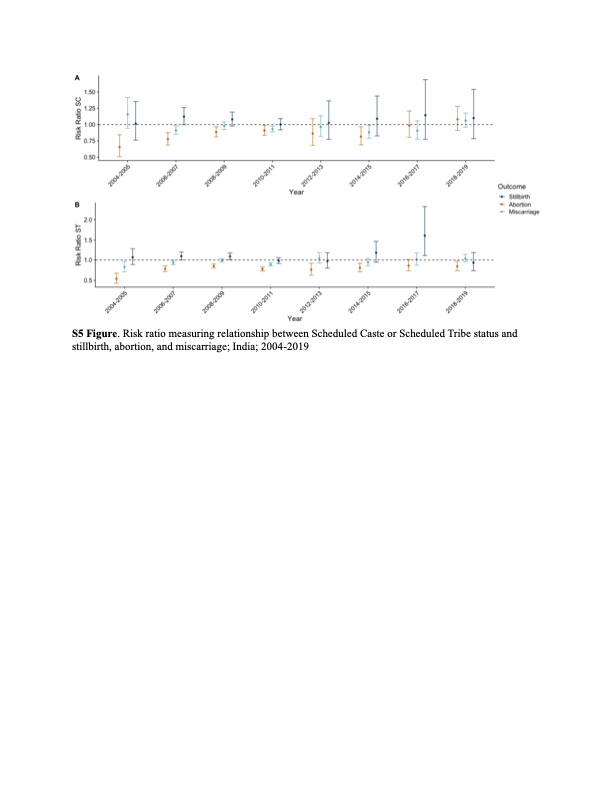

Supplement: S5 Fig — (TIFF) [file pgph.0003701.s006.tiff]
