# Supplementary material for: Socioeconomic inequalities in adverse pregnancy outcomes in India: 2004–2019
Source: PLOS Glob Public Health. 2024 Sep 18;4(9):e0003701. doi: 10.1371/journal.pgph.0003701 (PMC11410185; doi:10.1371/journal.pgph.0003701)
Supplement: S1 Text — (DOCX) [file pgph.0003701.s007.docx]

**S1 Text**

Creation of a Wealth Index

We adapted the approach applied by the DHS program for measuring asset-based household wealth to produce a household wealth index. We applied Principal Component Analysis (PCA). PCA is a data reduction technique used to reduce the dimensionality among correlated variables,[1] in this case by reducing items that measure household assets into a principal component or domain, or set of domains. The intuition is to identify the smallest number of components that explains substantial variation among the set of variables.[2] PCA works best when the variables are highly correlated, which is generally the case for socioeconomic indicators, including the asset measures used to generate the wealth index.[3] The DHS program uses the first principal component to estimate a continuous household wealth score for each household.[4]

To create the wealth index, the asset indices for all five surveys were compared. Variables that matched across the surveys were selected for creation of the index. These variables include whether the household owned a: radio; fridge; bicycle; motorcycle; animal cart; car; television; computer; or land. We also included: if the household drinking water is treated; whether the water source is from an improved facility; the source of water for the household; the type of toilet; if the toilet is shared; and the type of cooking fuel. Given substantial missing data on the mobile phone asset question in NFHS-5, mobile phone ownership was not included in the calculation of the wealth index. In contrast to the DHS approach of dichtomizing categorical variables, we followed recent guidance to order categorical variables sequentially by amount of resources used.[5] Ordering these categorical variables allows for greater discrimination among those of the lowest socioeconomic status.[6] For these categorical variables (e.g. type of toilet), the response items were matched and ordered according to the World Health Organization (WHO) criteria of improved vs. unimproved water and sanitation systems (**Table A**).[7] The binary variables 0 category included both missing and those who did not endorse the item.[8]

Once these items had been selected, squared multiple correlations was used to examine variation. Variables explaining less than 5% of the variance were dropped from the asset index; this included owning an animal cart and whether water source was improved. The remaining variables were included in a principal component analysis with polychoric correlation. Mean missing substitution was used to impute missing categorical variables with the mean value.[8] The associated eigenvalues of the PCA can be seen in **Figure A**. The first component explained 44% of the total variance of the included variables, and was therefore used to proxy household wealth. The continuous wealth score was used to rank each observation within the distribution of 0 – 1 for each survey year. Observations that had the same score were given the same rank. Scores were then divided into quintiles ranging from 0.2 (poorest) to 1 (richest) for descriptive figures.

**Table A**. Ordered items for asset index

| Variable | Ordered Items |
| --- | --- |
| Water Source | 1 piped water into dwelling yard/plot |
|  | 2 public tap stand pipe |
|  | 3 tube well or borehole |
|  | 4 protected dugwell |
|  | 5 tanker/truck/card |
|  | 6 unprotected dug well |
|  | 7 surface water  8 other |
| Improved Water | 1 no facilities |
|  | 2 unimproved facilities |
|  | 3 improved facilities |
| Toilet Type | 1 pour/flush latrine: connected to piped sewer system |
|  | 2 pour/flush latrine: connected to septic tank |
|  | 3 pour/flush latrine: connected to pit latrine |
|  | 4 pour/flush latrine: connected to something else |
|  | 5 pit latrine: ventilated improved pit |
|  | 6 pit latrine: with slab |
|  | 7 pit latrine: open or without slab |
|  | 8 service latrine /dry toilet |
|  | 9 open defecation/no facility/open space or field |
| Cooking Fuel | 1 electricity |
|  | 2 lpg/ natural gas |
|  | 3 biogas |
|  | 4 kerosene |
|  | 5 coal / ignite / charcoal |
|  | 6 wood |
|  | 7 straw / shrubs / grass / agricultural crop waste |
|  | 8 dung cakes |


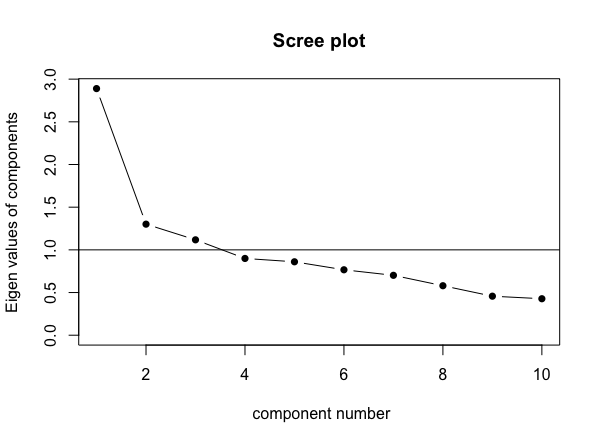


**Figure A.** Scree plot from PCA for wealth index creation

1. Lattin JM, Carroll JD, Green PE. Analyzing multivariate data. Pacific Grove, CA: Thomson Brooks/Cole; 2003. xxiv, 556 p. : ill. p.

2. Abdi H, Williams LJ. Principal component analysis. WIREs Computational Statistics. 2010;2(4):433-59.

3. Van Belle G, Fisher LD, Heagerty PJ, Lumley T. Biostatistics: a methodology for the health sciences: John Wiley & Sons; 2004.

4. Rutstein SO. Steps to constructing the new DHS Wealth Index. Rockville, MD: ICF International. 2015.

5. Martel P, Mbofana F, Cousens S. The polychoric dual-component wealth index as an alternative to the DHS index: Addressing the urban bias. J Glob Health. 2021;11:04003.

6. Poirier MJP, Grépin KA, Grignon M. Approaches and Alternatives to the Wealth Index to Measure Socioeconomic Status Using Survey Data: A Critical Interpretive Synthesis. Social Indicators Research. 2020;148(1):1-46.

7. Organization WH, Fund UNCs. JMP Methodology 2017 Update & SDG Baselines. WHO Geneva, Switzerland; 2018.

8. Lisa Hjelm, Astrid Mathiassen, Darryl Miller, Amit Wadhwa. VAM Guidance Paper: Creation of a Wealth Index. World Food Programme; 2017.
